# The “line-band closure” technique: a new endoscopic traction method for closure of a large defect

**DOI:** 10.1055/a-2452-5130

**Published:** 2024-11-13

**Authors:** Fei Liu, Zhenyun Gong, Duanmin Hu

**Affiliations:** 1 105860 Department of Gastroenterology, Second Affiliated Hospital of Soochow University, Suzhou, China

We describe the case of a 55-year-old woman with a solitary, yellow, subepithelial mass found in the large curvature of the stomach body on upper gastrointestinal endoscopy ( Fig. 1 **a** ). The lesion had a size of 8 × 6 mm and was considered a neuroendocrine tumor. Endoscopic submucosal dissection (ESD) was performed to achieve complete resection of the lesion. After the lesion was removed, a 20 × 15 mm defect was left ( Fig. 1 **b** ). In order to prevent delayed perforation and bleeding, the defect was closed.

**Fig. 1:**
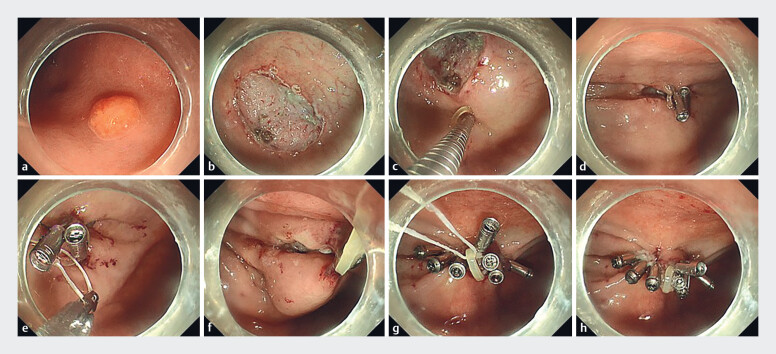
Endoscopic characteristics of the lesion and stages of the “line-band closure” technique. **a** A yellow subepithelial mass in the large curvature of the stomach body, with a size of 8 × 6 mm. **b** After the lesion was removed, a 20 × 15 mm defect was left. **c, d** The clip with a rubber band narrowed the distance between the two sides of the defect. **e** A clip was used to grasp the dental floss and pass it through the band. **f** By extracorporeal traction, the defect was pulled up to form a tent-like peak, and the two sides of the defect were brought closer together and linearized. **g** The defect was gradually closed from the traction point to both sides with clips. **h** After the defect was completely closed, the floss was pulled out.

The procedure for defect closure was as follows ( Fig. 1 **c–h** , Fig. 2 , Video 1 ). 1) The clip with an orthodontic rubber band (5 mm in diameter; Xufei, Hangzhou, China) was attached to one side of the defect, and a second clip clamped the band and was attached to the opposite side of the defect to narrow the distance between the two sides. 2) A third clip was used to grasp a length of dental floss and pass it through the band. By extracorporeal traction, the defect was pulled up to form a tent-like peak, and the two sides of the defect were brought closer together and linearized. The defect was gradually closed from the traction point to both sides with clips. 3) After the defect was completely closed, the floss was slowly pull out.

**Fig. 2:**
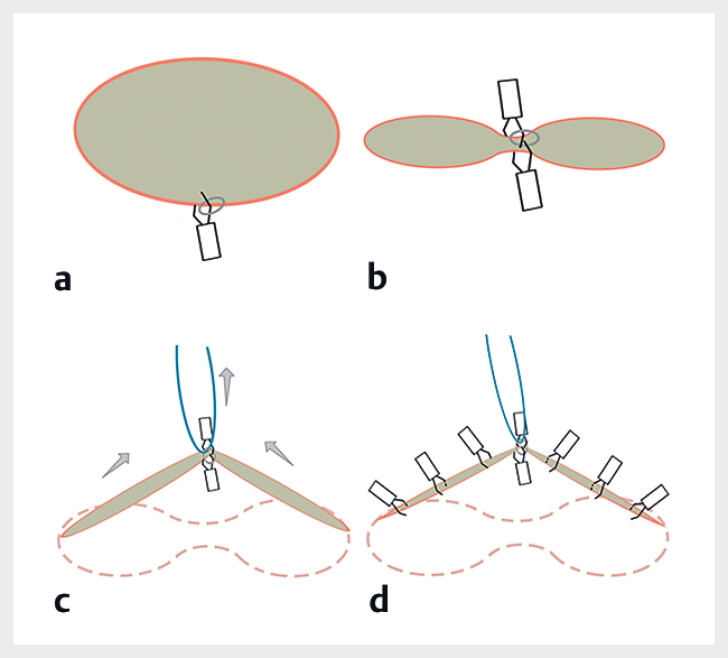
Illustration of the “line-band closure” technique.

The “line-band closure” technique.Video 1

Pathological examination revealed a neuroendocrine tumor (G1) with a negative margin. The patient had no adverse events and was discharged after 3 days.

The closure of large post-ESD mucosal defects is a challenge. Although new endoscopic closure techniques have sprung up in recent years, most of them require complex or specialized equipment and are technically challenging 1 2 3 4 . In this report, we used dental floss combined with a rubber band to apply extracorporeal traction on the defect. By traction, the defect forms a tent-like peak, bringing the two sides closer together and linearizing the pseudo-suture path. In addition, traction improves and maintains the endoscopic field of view, even if there is a large perforation in the gastrointestinal wall and the stomach cavity is difficult to fill. We call this technique “line-band closure,” and it is simple, feasible, and safe, and may represent a useful new endoscopic closure technique.

Endoscopy_UCTN_Code_TTT_1AO_2AO

## Notes

Endoscopy E-Videos https://eref.thieme.de/e-videos
*E-Videos* is an open access online section of the journal *Endoscopy* , reporting on interesting cases and new techniques in gastroenterological endoscopy. All papers include a high-quality video and are published with a Creative Commons CC-BY license. Endoscopy E-Videos qualify for HINARI discounts and waivers and eligibility is automatically checked during the submission process. We grant 100% waivers to articles whose corresponding authors are based in Group A countries and 50% waivers to those who are based in Group B countries as classified by Research4Life (see: https://www.research4life.org/access/eligibility/ ). This section has its own submission website at https://mc.manuscriptcentral.com/e-videos .
